# SOX combined with tislelizumab and low-dose radiation therapy for the neoadjuvant treatment of locally advanced gastric/gastroesophageal junction adenocarcinoma: study protocol for a prospective, multicenter, single-arm, phase Ib/II clinical trial

**DOI:** 10.3389/fimmu.2024.1431957

**Published:** 2024-11-13

**Authors:** Peng-Fei Zhang, Ye Chen, Wen-Ke Li, Zhu-Mei Luo, Ji Chen, Kun Qian, Xiao-Dong Chen, Mo-Jin Wang, Ming Liu

**Affiliations:** ^1^ Gastric Cancer Center, Division of Medical Oncology, Cancer Center, West China Hospital, Sichuan University, Chengdu, China; ^2^ Abdominal Oncology Ward, Cancer Center, West China Hospital, Sichuan University, Chengdu, China; ^3^ Department of Medical Oncology, Chengdu Third People’s Hospital, Chengdu, China; ^4^ Department of Medical Oncology, Chengdu Seventh People’s Hospital, Chengdu, China; ^5^ Department of General Surgery, The First Affiliated Hospital of Chongqing Medical University, Chongqing, China; ^6^ Department of General Surgery, Sichuan Cancer Hospital & Institute, Sichuan Cancer Center, Affiliate Cancer Hospital of University of Electronic Science and Technology of China (UESTC), Chengdu, China; ^7^ Department of General Surgery & Laboratory of Gastric Cancer, State Key Laboratory of Biotherapy/Collaborative Innovation Center of Biotherapy and Cancer Center, West China Hospital, Sichuan University, Chengdu, China; ^8^ Gastric Cancer Center, West China Hospital, Sichuan University, Chengdu, China

**Keywords:** gastric cancer, neoadjuvant therapy, S-1, oxaliplatin, tislelizumab, low-dose radiation therapy

## Abstract

**Background:**

Recently, the clinical benefits of neoadjuvant chemotherapy combined with immunotherapy have been observed in patients with locally advanced gastric or gastroesophageal junction (G/GEJ) cancer; however, the pathological complete response (pCR) and long-term survival rates are still unsatisfactory. The aim of this study is to investigate the efficacy and safety of chemotherapy combined with tislelizumab and low-dose radiation therapy (LDRT) for the neoadjuvant treatment of locally advanced G/GEJ cancer.

**Methods:**

This is a prospective, multicenter, single-arm, phase Ib/II trial. In the phase Ib study, 5 patients will be enrolled in each treatment group with different radiation doses. In the phase II study, a total of 44 patients will be enrolled. Eligible patients will be registered and receive three cycles of SOX regimen chemotherapy (S-1: 40-60 mg Bid, d1-14, q3w; oxaliplatin: 130 mg/m^2^, iv drip, d1, q3w) plus tislelizumab (200 mg, iv drip, d1, q3w). Simultaneously, LDRT will be planned and administered after the first cycle of systemic therapy. Radical D2 gastrectomy will be performed 4-6 weeks after the last administration of chemotherapy plus tislelizumab. The primary endpoint of phase Ib study is to determine the optimal radiation dose for phase II study. The primary endpoint of phase II is the pCR rate. The secondary endpoints include R0 resection rate, major pathological response (MPR) rate, 2-year event-free survival (EFS) rate, 2-year overall survival (OS) rate and safety profile. Moreover, we will also explore potential molecular markers for predicting the benefit and safety of this neoadjuvant regimen. Written informed consent should be provided by all patients enrolled in the study. The study protocol was approved by the independent ethics committee at each institution.

**Discussion:**

This is the first study to explore the efficacy and safety of neoadjuvant chemotherapy combined with tislelizumab and LDRT in G/GEJ cancer patients, the results of which may provide novel treatment strategy for patients with locally advanced G/GEJ adenocarcinoma.

**Clinical trial registration:**

ClinicalTrials.Gov, identifier NCT06266871.

## Introduction

Gastric/gastroesophageal junction (G/GEJ) cancer remains one of the most common malignant tumors and the fourth leading cause of cancer-related deaths. It is estimated that more than one million new cases and more than 7 hundred thousand deaths from G/GEJ cancer occurred in 2020 worldwide (1). Currently, radical gastrectomy still represents the main curative treatment for G/GEJ cancer; however, the risk of disease recurrence or metastasis is still high in patients undergoing gastrectomy, especially those with locally advanced diseases (2). Neoadjuvant therapy has been demonstrated to significantly decrease the tumor stage, improve the pathological complete response (pCR) rate, increase the rate of R0 resection, and reduce the risk of postoperative recurrence or metastasis in a variety of cancers (3–5). Recently, a number of studies, including the MAGIC study in the UK, the PRODIGY study in South Korea, and the RESONANCE and RESOLVE studies in China, have demonstrated that neoadjuvant chemotherapy represents a promising regimen to improve the pCR rate and prolong survival of G/GEJ cancer patients (6–9). Moreover, the therapeutic benefits of preoperative concurrent chemoradiotherapy, have also been confirmed by the CROSS and POET studies (10, 11). Therefore, chemotherapy or chemoradiotherapy has become the standard neoadjuvant regimen for locally advanced G/GEJ cancer and has been recommended by a series of treatment guidelines (12–14). Despite the clinical benefits of neoadjuvant chemotherapy or chemoradiotherapy, the pCR and long-term survival rates are far from satisfactory, and the perioperative treatment for locally advanced G/GEJ cancer still needs further optimization.

Currently, the treatment for advanced G/GEJ cancer has entered the era of immunotherapy, and the combination of immune checkpoint inhibitors (ICIs) and chemotherapy has been demonstrated to prolong overall survival (OS) and progression-free survival (PFS) compared to chemotherapy alone in the first-line setting of advanced or metastatic G/GEJ cancer patients (15, 16). These promising results have sparked the interest of researchers to further investigate the wide application of ICIs plus chemotherapy in the neoadjuvant setting for locally advanced G/GEJ cancer. In a single-arm, phase II clinical study conducted by Jiang et al., 36 patients with locally advanced, resectable G/GEJ cancer were enrolled to receive sintilimab plus XELOX regimen. PCR and R0 resection rates were 19.4% and 97.2%, respectively (17). Another study also showed similar results. The pCR rate in patients with locally advanced G/GEJ adenocarcinoma treated with the combination of tislelizumab and SOX was 25%, and the annual recurrence-free survival (RFS) and OS were 90.0% and 91.4%, respectively (18). In the phase 3, randomized Keynote 585 trial, 402 were assigned to the pembrolizumab plus cisplatin-based chemotherapy group and 402 to the placebo plus cisplatin-based chemotherapy group. After median follow-up of 47.7 months, pembrolizumab was superior to placebo for pCR (12.9% (95% CI 9.8-16.6) vs 2.0% (0.9-3.9); p<0.00001) (19). Another global, phase 3, randomized, double-blind MATTERHORN study also assessed perioperative durvalumab (D) with FLOT in patients with resectable G/GEJC cancer. A statistically significant improvement in pCR was observed with addition of D to FLOT versus FLOT (19% vs 7%; p<0.00001) (20). These data suggest that the combination of chemotherapy and immunotherapy is a promising treatment strategy for the neoadjuvant treatment of locally advanced G/GEJ cancer.

Moreover, increasing evidence has shown a synergistic sensitization effect between radiotherapy and programmed cell death protein 1 (PD-1) inhibitors (21–24). In the Neo-PLANET study, the safety and efficacy of the combination of camrelizumab and chemoradiation in neoadjuvant therapy for locally advanced G/GEJ cancer were evaluated. The R0 resection, pCR and major pathological response (MPR) rates were 91.7%, 33.3% and 41.7%, respectively (25). In another similar study, 34 patients with locally advanced G/GEJ cancer were included and received sintilimab combined with concurrent chemoradiation. Among these patients, 13 achieved pCR (38.2%), and 27 achieved MPR (79.4%) (26). Although it has significantly greater efficacy, the combination of neoadjuvant radiotherapy may increase the incidence of adverse events (AEs), increase the difficulty of surgery and delay wound healing. For example, approximately 15.2% of patients experienced delayed surgery due to severe AEs in the Neo-PLANET study. In the past decade, low-dose radiation therapy (LDRT) has been shown to promote the infiltration of immune cells into the tumor microenvironment (TME) and turn “cold tumors” into “hot tumors”, thereby enhancing the efficacy of immunotherapy (27). LDRT induces the release of dsDNA and damage-associated molecular patterns (DAMPs) in tumor cells and promotes the activation of the innate immune system. LDRT can also increase the tumor antigen presentation ability of DCs. Moreover, LDRT upregulates the expression of MHC class I and PD-L1 in tumor cells, which promotes antigen recognition by T cells (28). In addition, LDRT can also significantly improve patient tolerance to treatment, reduce surgical complications, and decrease patient economic burden, which represents a promising treatment strategy for patients with cancer. In a study, Yin et al. have investigated the effect of triple treatment consisting of hypofractionated radiation therapy (HFRT), ICI, and LDRT in mice and patients with non-small cell lung cancer (NSCLC) (29). In bilateral mouse tumor models, HFRT treatment of the primary tumor combined with LDRT treatment of the abscopal tumor and anti-PD1 therapy enhances the response compared with HFRT/anti-PD1, HFRT/LDRT, or LDRT/anti-PD1 treatments and complete response was observed in more than half of the mice treated with triple therapy. In 9 patients treated with this triple therapy, 3 and 2 patients showed PR and SD. In another study, Zhou et al. conducted a prospective phase 1 study to explore the safety and tolerability of LDRT and stereotactic body radiotherapy (SBRT) plus sintilimab as first-line treatment for stage IV PD-L1+ NSCLC patient (30). 29 patients were enrolled. In the dose-escalation phase, patients received SBRT (30Gy/3f) to small lesions and LDRT (2 Gy/1f, 4 Gy/2f, or 10 Gy/5f) to a large lesion concurrently, followed by sintilimab. No dose-limiting toxicities (DLT) were observed during the dose-escalation phase and 4 Gy/2f was the recommended LDRT dose. After a median follow up of 15.6 months, ORR and confirmed ORR were 60.7% and 57.1%, respectively. Median PFS was 8.6 months (95% confidence interval 3.7-16.5), and median OS was not reached. All these data suggested that LDRT could significantly enhance the efficacy of ICIs and may be a promising strategy in the neoadjuvant setting of advanced G/GEJ cancer.

In this study, we will explore the efficacy and safety of chemotherapy combined with tislelizumab and LDRT in the neoadjuvant treatment of locally advanced G/GEJ cancer.

## Methods and analysis

**Figure 1:**
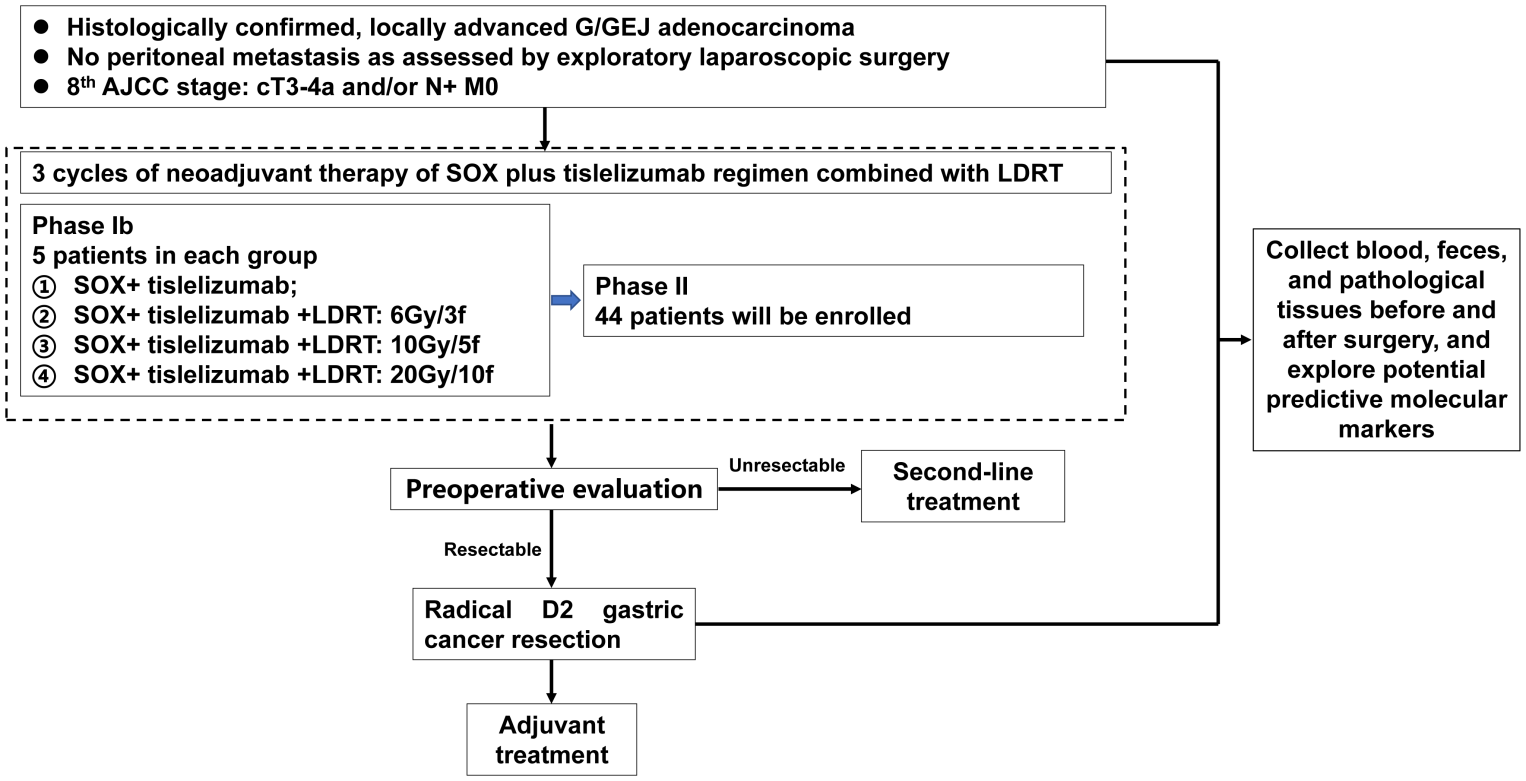
Study flow chart.

This prospective, multicenter, single-arm, phase Ib/II trial was designed to assess the efficacy and safety of chemotherapy combined with tislelizumab and LDRT for the neoadjuvant treatment of locally advanced G/GEJ cancer. The study protocol and the informed consent forms have been reviewed and approved by institutional academic review committees and ethics committees at each institution. The study will be performed in accordance with the Declaration of Helsinki and Good Clinical Practice Guidelines. All subjects enrolled in the study should provide written informed consent and all data are handled confidentially. The study is prospectively registered at ClinicalTrials.Gov (NCT06266871). A schematic diagram of the study design is shown in **Figure 1**.

### Endpoints

The primary endpoint of phase Ib study is to determine the optimal radiation dose for phase II study by evaluating the safety and tolerability of the SOX regimen combined with tislelizumab and different doses of LDRT (6 Gy/3 fractions, 10 Gy/5 fractions and 20 Gy/10 fractions).

The primary endpoint of phase II is the pCR rate, which is defined as the absence of viable tumor cells assessed by histological evaluation criteria after neoadjuvant therapy. The secondary endpoints include the R0 resection rate (defined as the rate of the complete surgical removal of any residual cancer cells in the tumor bed), MPR rate (defined as tumor residual cells ≤10% in the surgical specimen), the 2-year event-free survival (EFS) rate, the 2-year OS rate and the safety profile. AEs will be recorded and evaluated according to the National Cancer Institute’s Common Terminology Criteria for Adverse Events (NCI CTCAE, version 5.0). Serious adverse events (SAEs) are defined as death, hospitalization or prolonged hospitalization, permanent or severe disability, teratogenesis, or other significant clinical sequelae. The exploratory endpoints include predictive biomarkers of immunotherapy response in tumor tissue, peripheral blood and feces.

### Study population and eligibility criteria

The key inclusion criteria include the following:

1. Age 18-75 years.
2. Histologically or cytologically confirmed diagnosis of locally advanced G/GEJ adenocarcinoma (cT3-T4a N+ M0) as assessed by exploratory laparoscopic surgery, ultrasonography and/or CT/MRI.
3. Resectable G/GEJ cancer, as assessed by experienced surgeons.
4. Eastern Cooperative Oncology Group performance score (ECOG PS) ≤1.
5. Agree to provide blood, feces, and tissue specimens.
6. The expected survival is longer than 3 months.
7. There was no previous antitumor treatment (including chemotherapy, radiotherapy, targeted therapy, immunotherapy, interventional therapy, or other treatments with antitumor effects).
8. Adequate organ function including the following:
  • Total bilirubin ≤1.0 times the upper limit of normal (ULN);
  • Aspartate transaminase (AST) and alanine transaminase (ALT) ≤2.5×ULN;
  • Serum creatinine ≤ 1.0×ULN;
  • Platelet count ≥ 100,000/mm^3^;
  • Hemoglobin (Hb) ≥ 9 g/dL;
  • Absolute neutrophil count (ANC) ≥ 1500/mm^3^;
9. Strict contraception.
10. Patients must be able to understand and be willing to sign the written informed consent form. A signed informed consent form must be obtained prior to the conduct of any trial-specific procedure.

The key exclusion criteria include were as follows:

1. Patients who were undergoing other drug clinical trials or who had participated in any drug clinical trials one month before enrollment.
2. Other tumors that have not been treated or exist at the same time, except carcinoma *in situ* of the cervix, cured basal cell carcinoma or superficial bladder tumor. If the tumor was cured and no evidence of disease was found for more than 3 years, the patient could be enrolled. All other tumors must be treated for at least 3 years before enrollment.
3. Allergy to oxaliplatin, S-1, tislelizumab and similar drugs, or suspected allergies.
4. Serious gastrointestinal diseases, such as chronic inflammatory bowel disease, can affect the absorption of oral chemotherapy drugs.
5. History of gastric perforation within 6 months.
6. Clinical manifestations include significant arrhythmia, myocardial ischemia, severe atrioventricular block, heart failure, and severe heart valve disease; severe lung function impairment; impaired blood system, liver and kidney function who cannot tolerate radiotherapy and chemotherapy; severe bone marrow failure; uncontrollable infections.
7. With uncontrollable mental illness.
8. Pregnant or lactating women.
9. Unable to comply with the research program or procedures.

### Intervention

Laparoscopic exploration is required for all patients to detect occult peritoneal metastases. Patients who meet the eligibility criteria will be enrolled and signed the informed consent form. All patients will start one cycle of neoadjuvant therapy with SOX plus tislelizumab: S-1: 40-60 mg Bid, d1-14, q3w; oxaliplatin: 130 mg/m^2^, iv drip, d1, q3w; tislelizumab: 200 mg, iv drip, d1, q3w. Then, LDRT will be performed in the target area (including the primary gastric lesion and positive/suspected positive lymph nodes). After radiotherapy, patients will receive another two cycles of the SOX plus tislelizumab regimen. Radical D2 gastric cancer resection will be performed 4-6 weeks after the last administration of the SOX plus tislelizumab regimen. Adjuvant therapy will start in 4-6 weeks after surgery, and we recommend adjuvant treatment with SOX regimen for up to 5 cycles.

### Statistical analysis

In the phase Ib study, 5 patients will be enrolled in each treatment group, with a total of 20 patients in 4 treatment groups. In the phase II study, the sample size was calculated based on the assumption that the pCR rate of patients receiving neoadjuvant SOX chemotherapy plus tislelizumab was 16%. A total of 40 patients treated with the neoadjuvant SOX regimen combined with tislelizumab and LDRT will provide 80% power to achieve a pCR rate of 30% at a one-sided 10% alpha level. Considering a 10% drop out rate, 44 assessable patients will be enrolled in the study. Descriptive statistics of baseline and clinicopathological characteristics will be performed. The pCR rate, MPR rate, R0 resection rate will be calculated, and the corresponding confidence intervals (CIs) will be estimated by Blaker’s binomial exact method. Kaplan-Meier estimates of EFS and OS probabilities will also be determined, with respective 95% CIs. The primary analyses will be performed in the intention-to-treat (ITT) population. All AEs will be analyzed in the safety population, defined as patients who receive at least one dose of neoadjuvant treatment. Neoadjuvant or adjuvant treatment-related emergent AEs will be reported separately due to the different regimens applied. Surgery-related morbidity and mortality will be analyzed in the per-protocol population, defined as patients who are compliant with the study protocol and proceed to surgery. To avoid the occurrence of missing data, we strived to improve the design of this clinical study and collected and managed raw data scientifically. Multiple imputation will be used to impute missing outcome data for patients who withdrew or were lost to follow-up.

## Discussion

Currently, neoadjuvant chemotherapy is recommended by a number of treatment guidelines and is widely applied to the treatment of patients with locally advanced G/GEJ cancer (12–14). However, the pCR rate of G/GEJ cancer patients treated with neoadjuvant chemotherapy is still low, and novel neoadjuvant strategies are urgently needed. Chemotherapy combined with PD-1 inhibitors has been demonstrated to prolong OS and PFS compared to chemotherapy alone in the first-line setting of advanced or metastatic G/GEJ cancer patients, which is recommended as the standard treatment option for patients with advanced or metastatic G/GEJ cancer (15, 16). Recently, the efficacy and safety of chemotherapy combined with PD-1 inhibitors in the neoadjuvant setting for locally advanced G/GEJ cancer patients have also been evaluated in a series of studies (17, 18). Despite the higher pCR rate and R0 resection rate, these benefits did not translate to longer survival (31). To further improve the effectiveness of neoadjuvant therapy, combination with other treatment strategies, including other immune checkpoint inhibitors, radiotherapy, and antiangiogenic agents, is necessary.

The role of immunotherapy plus concurrent chemoradiotherapy (cCRT) in the perioperative treatment of G/GEJ cancers has been evaluated in recent years. The Neo-PLANET study revealed a pCR rate of 33.3% (95% CI, 18.6–51.0), an MPR rate of 44.4% (95% CI, 27.9–61.9), and an R0 resection rate of 91.7% in 36 patients with resectable T3-4 N+M0 adenocarcinoma of the stomach or GEJ, which indicated that the combination of camrelizumab and cCRT is a neoadjuvant therapy for locally advanced gastric adenocarcinoma. In another similar study, the efficacy and safety of neoadjuvant sintilimab and cCRT were also investigated in patients with locally advanced G/GEJ cancers, which resulted in a pCR rate of 38.2% and an MPR rate of 79.4%. However, the combination of neoadjuvant radiotherapy may increase the incidence of AEs. For example, in a single-arm, phase II clinical study evaluating the efficacy and safety of sintilimab plus the XELOX regimen in patients with locally advanced, resectable G/GEJ cancer, ten (27.8%) patients experienced grade 3 TRAEs. However, in the study of patients treated with sintilimab and cCRT, the rate of grade 3/4 AEs in the neoadjuvant period was 50.0%.

Recently, a growing number of studies have focused on the effect of LDRT on cancer immunotherapy. Preclinical studies have shown that LDRT can reprogram the tumor immune microenvironment by transiently inflaming tumors, inducing macrophage M1 polarization, improving the tumor antigen presentation ability of DCs, and upregulating the expression of MHC class I and PD-L1 in tumor cells (27, 28). In a *post hoc* analysis, the efficacy of the combination of stereotactic ablative RT and immunotherapy was investigated. The ORR of low-dose lesions was much greater than that of no-dose lesions (58% vs 18%) (32). In the RACIN study, LDRT combined with low-dose cyclophosphamide, nivolumab, ipilimumab, and aspirin resulted in an 87.5% disease control rate in heavily treated patients. All these results indicate that LDRT combined with immunotherapy is a promising treatment strategy (27).

Despite its promising therapeutic effect, potential risks associated with combining immunotherapy and LDRT should also be addressed. First, LDRT may also cause skin and mucosal injury, bone marrow suppression, and other conventional AEs (26). In addition, LDRT may also cause several special AEs, especially when applied to digestive organs. In the past few years, several studies have demonstrated that preoperative chemoradiotherapy may increase the risk of postoperative anastomotic leakage and stenosis (33–37). Although the radiation dose of LDRT is significantly lower compared to traditional radiotherapy, potential risk of postoperative anastomotic leakage and stenosis is still high, which is needed to be closely monitored during the research process.

In conclusion, we conducted a prospective, single-arm, phase Ib/II trial to investigate the efficacy and safety of chemotherapy combined with tislelizumab and LDRT for the neoadjuvant treatment of locally advanced G/GEJ cancer. To the best of our knowledge, this is the first study to explore the efficacy of neoadjuvant chemotherapy combined with tislelizumab and LDRT in G/GEJ cancer patients, which will provide a novel treatment strategy for patients with locally advanced G/GEJ adenocarcinoma.

## Data Availability

The original contributions presented in the study are included in the article/supplementary materials, further inquiries can be directed to the corresponding author/s.
